# Genome Sequence of *Madurella mycetomatis* mm55, Isolated from a Human Mycetoma Case in Sudan

**DOI:** 10.1128/genomeA.00418-16

**Published:** 2016-05-26

**Authors:** Sandra Smit, Martijn F. L. Derks, Sander Bervoets, Ahmed Fahal, Willem van Leeuwen, Alex van Belkum, Wendy W. J. van de Sande

**Affiliations:** a Department of Medical Microbiology and Infectious Diseases, Erasmus MC, Rotterdam, The Netherlands; b Bioinformatics Group, Wageningen University, Wageningen, The Netherlands; c ServiceXS B.V., Leiden, The Netherlands; d Mycetoma Research Centre, University of Khartoum, Khartoum, Sudan; e Professorate Innovative Molecular Diagnostics, University of Applied Sciences Leiden, Leiden, The Netherlands; f Microbiology R&D Clinical Unit, bioMérieux, La Balme les Grottes, France

## Abstract

We present the first genome sequence for a strain of the main mycetoma causative agent, *Madurella mycetomatis*. This 36.7-Mb genome sequence will offer new insights into the pathogenesis of mycetoma, and it will contribute to the development of better therapies for this neglected tropical disease.

## GENOME ANNOUNCEMENT

Mycetoma is a chronic granulomatous subcutaneous tropical infectious disease, caused by either fungi (eumycetoma) or bacteria (actinomycetoma). The most common causative agent of human mycetoma is the fungus *Madurella mycetomatis* (1). A characteristic of mycetoma is that the causative agents organize themselves in grains, which are black in the case of *M. mycetomatis*. The pathogenesis of mycetoma is barely understood. In recent years, genome sequences of the actinomycetoma causative agents, *Nocardia brasiliensis* (2, 3), *Streptomyces somaliensis* (4), and *Actinomadura madurae* (5) have been published, but no genome sequence for a fungal mycetoma agent was available until now. Here, we report the first genome sequence for a clinical isolate of *M. mycetomatis*.

*M. mycetomatis* mm55 was isolated on 25 November 1999 in the Mycetoma Research Centre (Khartoum, Sudan) from an extensive mycetoma case in the foot in a 22-year-old male patient. The patient from central Sudan had been suffering from mycetoma for more than 12 years. This strain was isolated by direct culture of the black grains obtained by a deep biopsy and identified by morphology, PCR-RFLP, and sequencing of the ITS region (6). Strain mm55 was kept on Sabouraud agar and ground in a mortar and pestle in liquid nitrogen, and the DNA was isolated with the Promega Wizard kit (Promega, Leiden, The Netherlands). The genome was reconstructed from Roche 454 (12.3× coverage), Illumina (28.2×), and PacBio (3.0×) data. Minimus2 (7) was used to merge one assembly made by the whole-genome sequencing assembler (8) and PBjelly (9), and another made by SPAdes (10) and SSPACE-LongRead (11). The resulting draft genome is 36.7 Mbp long, fragmented into 804 scaffolds (*N*_50_ of 81.8 kb; G+C content of 54.9%).

The genome was annotated using MAKER2 (12) and EvidenceModeler (13) (predictions with long introns were removed). Gene predictors SNAP and Augustus were trained using *Chaetomium globosum*. Protein and expressed sequence tag sequences of eight fungi (six in the order of *Sordariales*) were used as homology evidence. Functional annotation was done using BLASTp (14) against the Swiss-Prot and TrEMBL databases (15). Protein domains, Pfam superfamily IDs, and gene-ontology terms were assigned using InterProScan (16). The draft genome contains 10,707 protein-coding genes. CEGMA (17) and BUSCO (18) showed that 89% of core eukaryotic genes are completely present in the assembly, and OrthoFinder (19) indicated that 93% of the predicted genes have orthologs in other species.

The genomic data generated provide a potential leap forward in our comprehension of the biology and pathogenic mechanisms underlying human eumycetoma and will help our understanding of the formation of fungal grains. By comparing the genome to closely related fungi not causing mycetoma, proteins can be identified that could play a role in the development of the mycetoma grain. Furthermore, this sequence offers a rich resource for the identification of *M. mycetomatis*–specific proteins, which could be used to develop novel diagnostic tools or which could serve as novel drug targets. The latter is especially important, since the current treatment is often limited to azole treatment and amputation.

### Nucleotide sequence accession numbers.

This annotated genome (version 2.0) has been deposited at DDBJ/EMBL/GenBank under the accession number LCTW00000000, BioProject PRJNA267680.
